# Enhancing Rural Breast Cancer Screening Uptake: A Three‐Arm Comparative Study of Outreach Strategies

**DOI:** 10.1155/ijbc/4070359

**Published:** 2026-04-10

**Authors:** Komal Verma Saluja, Sangeeta Saxena, Mohammed Shadab Gouri, Priyanka Sangar, Sakshi Sharma, Harshvardhan Khokhar, Reshma Reja

**Affiliations:** ^1^ Department of Medicine and Radiology, Government Medical College, Kota, Kota, India, calicutmedicalcollege.ac.in; ^2^ Department of Radiology, Government Medical College, Kota, Kota, India, India, calicutmedicalcollege.ac.in; ^3^ Department of Community Medicine, Government Medical College, Kota, Kota, India, India, calicutmedicalcollege.ac.in; ^4^ Department of Medicine, Government Medical College, Kota, Kota, India, India, calicutmedicalcollege.ac.in

**Keywords:** awareness, breast cancer, community-based intervention, early detection, mobile screening unit, rural health, screening uptake, stigma reduction, women′s health

## Abstract

**Background:**

Breast cancer is the most prevalent cancer among women, with a notably high mortality‐to‐incidence ratio, especially in rural areas. Limited awareness and inadequate access to healthcare are major contributors to the high breast cancer mortality rate in rural women, often resulting from insufficient screening, delayed diagnosis, and treatment.

**Objectives:**

This study is aimed at evaluating the effectiveness of three distinct intervention strategies—medical personnel–led education, breast health volunteers, and mobile screening mammography van visits—in improving breast cancer awareness, increasing screening participation, and reducing associated stigmatization among rural women.

**Methods:**

A comparative, community‐based interventional study was conducted in three villages in neighboring districts. Rural communities were randomly allocated to one of the three intervention strategies. Baseline and postintervention data were collected using validated structured questionnaires assessing breast cancer knowledge, screening practices, stigma, and perceived barriers.

**Results:**

A total of 944 women aged 30–70 years were enrolled in the study. Overall, 63.7% of participants underwent screening. The mobile screening mammography van group achieved the highest screening uptake (68.7%) and the greatest increase in awareness scores, whereas the breast health volunteer–led group showed the most significant reduction in stigma. The medical personnel–led group, though cost‐effective in raising awareness, recorded lower screening participation and reported higher perceived barriers to screening mammography (e.g., transport, time, and financial constraints).

**Conclusion:**

The findings demonstrate that integrated, culturally tailored interventions, particularly those combining community engagement with accessible on‐site screening services, can effectively improve breast cancer awareness and screening uptake among rural women. Scaling up such multicomponent strategies may help overcome logistical and sociocultural barriers, ultimately contributing to earlier detection and reduced mortality.

## 1. Introduction

Breast cancer has emerged as the most prevalent cancer among women in our country, accounting for approximately 27% of all cancer cases in the female population [1, 2]. A particularly concerning aspect of this trend is the markedly high mortality‐to‐incidence ratio [1, 3]. The situation is especially dire in rural areas, where prevalence is rapidly increasing due to limited awareness and inadequate access to healthcare services [2, 3]. This high breast cancer mortality rate is largely attributed to late‐stage diagnoses, often resulting from insufficient screening [4]. Studies have consistently reported critically low screening uptake among rural women, leading to advanced‐stage presentations and poorer prognoses [4].

Early detection through regular screening is the cornerstone of effective cancer control and is strongly associated with improved survival outcomes, yet several barriers hinder its widespread adoption in rural areas [5, 6]. Multiple lines of evidence ranging from observational studies to randomized controlled trials demonstrate that organized screening programs for breast, cervical, and colorectal cancers are associated with significant reductions in disease‐specific mortality [7]. In the case of breast cancer, population‐based mammography screening has been associated with a 25%–31% reduction in mortality, emphasizing the clinical value of timely diagnosis [7]. Furthermore, engagement in screening programs is associated with a lower incidence of advanced‐stage and fatal breast cancers [8].

Multiple barriers impede effective breast cancer screening in rural communities. Cognitive barriers, such as a lack of awareness about available screening methods, are widespread, with over 84% of women reporting “do not know” as their reason for not undergoing screening [9]. Sociocultural factors, including stigma, health illiteracy, misconceptions, and discontinuous healthcare access, further complicate efforts to implement cost‐effective screening strategies [10, 11]. Knowledge gaps are evident, with studies indicating that while two‐thirds of rural women have heard of breast cancer, less than 7% are familiar with breast self‐examination (BSE), and the majority cannot identify any symptoms or risk factors [5, 6].

To address these challenges, targeted interventions aimed at enhancing awareness and improving access to screening services are essential. Globally, various initiatives have been implemented to promote breast cancer screening, including patient education, personalized reminders, patient navigation, financial incentives, and the use of community health workers [7, 12, 13]. Mobile screening units (MSUs) have also proven effective in overcoming logistical and structural barriers, particularly in underserved areas [7]. Multicomponent interventions that combine community demand (education) and community access (reducing financial and structural barriers) have been recommended by the Community Preventive Services Task Force [7, 14]. Such strategies have shown promise in increasing screening uptake in diverse populations, particularly when tailored to the cultural context of the target community [14].

This study is aimed at evaluating the effectiveness of three distinct strategies—medical personnel–led interventions, breast health volunteer (BHV)–led interventions, and mobile screening mammography van visits—in increasing breast cancer awareness and screening participation among women in rural areas of India. By assessing the reach, acceptability, and impact of these interventions, the study seeks to inform the development of scalable, cost‐effective, and culturally congruent models for early detection and mortality reduction in similar low‐resource settings.

## 2. Methods

### 2.1. Study Design and Ethical Considerations

This was a cluster‐randomized community‐based interventional study to evaluate the effectiveness of three different strategies for increasing breast cancer awareness and screening participation in rural areas. Ethical approval for the study was obtained from the Institutional Ethics Committee. Informed consent was obtained from all participants or an appropriate representative on behalf of illiterate participants, prior to study enrollment. Data confidentiality was maintained by anonymizing all collected information. Participants were fully informed about the purpose of the study, procedures, potential risks, and their right to withdraw at any time.

### 2.2. Study Setting

The study was conducted across three rural villages in neighboring districts, with sufficient geographic separation, designated as Villages A, B, and C, randomly selected from all the villages of these districts. All three villages were evaluated before the start of the study for comparable healthcare access, female literacy rates, prior screening program exposure, demographic profiles, and socioeconomic characteristics, ensuring that the clusters were reasonably balanced at the start of the study.

These rural sites were randomly selected due to their limited access to healthcare infrastructure in rural areas and the observed low baseline awareness regarding breast cancer and its screening modalities. The selected villages were then randomly allocated to one of three intervention arms using a computer‐generated random number sequence.

### 2.3. Study Population

Women aged 30–70 years residing in the selected rural areas were eligible to participate. Inclusion criteria included willingness to provide informed consent and the ability to attend awareness sessions or participate in community education programs. Women with a history of breast cancer, those currently undergoing treatment for any malignancy, individuals with severe physical or mental illnesses, nonresidents, pregnant women, or those unwilling to consent were excluded from participation.

### 2.4. Sample Size Calculation

The sample size was determined based on expected screening uptake rates. It was estimated that the medical personnel–led intervention group would achieve a screening uptake of 40%, whereas the BHV‐led intervention group was expected to yield an uptake of 25%. Using these projected proportions, and assuming a two‐sided alpha of 0.05 and 80% power (*β* = 0.20), the minimum required sample size was estimated to be 144 participants per arm to detect a statistically significant difference. To account for potential attrition, the final sample size was inflated to 180 participants per group.

### 2.5. Intervention Strategies

**Figure 1:**
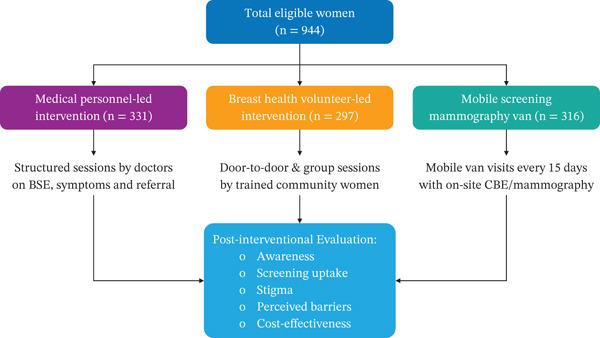
Study design and flow. Random assignment of each village to one intervention, an implementation phase of 24 months followed by outcome evaluation.

Three distinct breast health intervention models were evaluated to enhance awareness and participation in breast cancer screening. These interventions included medical personnel–led health education intervention, community‐based BHV‐led intervention, and mobile screening mammography van intervention. Villages were randomly assigned to one of the three intervention arms to minimize selection bias and ensure comparability across groups (Figure 1). Each intervention was implemented for 24 months, followed by outcome evaluation.

#### 2.5.1. Medical Personnel–Led Health Education Intervention

Trained healthcare professionals delivered structured educational sessions using audiovisual aids. The sessions emphasized recognition of breast cancer symptoms, early detection methods, BSE, and the importance of regular screening. Information on the availability and accessibility of mammography services was also provided. Printed materials, including leaflets and posters, were distributed to reinforce messages. Each participant was invited to attend two sessions (40–45 min each). A total of 12 sessions were conducted, with an average attendance rate of 63.0%.

#### 2.5.2. BHV‐Led Intervention

Literate, community‐based female volunteers were recruited and trained by medical personnel in breast health aspects. These BHVs carried out door‐to‐door outreach, individualized counseling, and small‐group discussions to disseminate information, encourage screening, and address cultural barriers and stigma. This grassroots approach was designed to ensure cultural sensitivity and foster community engagement. Each volunteer covered approximately 20 households. In total, 24 group sessions of 30–45 min were organized, with an average of 12 participants per session (average attendance rate of 79%).

#### 2.5.3. Mobile Screening Mammography Van Intervention

A mobile van equipped for clinical breast examination (CBE) and digital mammography visited the target village quarterly. Staffed by trained healthcare professionals, the van provided on‐site screening and referred women for further evaluation when abnormalities were detected. Over the 24‐month period, eight visits were completed, achieving a screening coverage rate of 68.7%.

Across all three arms, participant engagement, fidelity of implementation, and coverage metrics were systematically monitored to ensure program quality, consistency, and effectiveness.

### 2.6. Questionnaire Instruments

Breast cancer awareness was assessed before and after the intervention using a validated structured questionnaire (BCAM [Breast Cancer Awareness Measure]) designed to measure knowledge of risk factors, warning signs, screening practices, and age perceptions [15]. The pooled awareness score was calculated by summing correct responses across four domains: warning signs, risk factors, screening practices, and breast cancer–related age perceptions. Rescaled scores ranged from 0 to 10, with higher scores indicating greater awareness. Stigma related to breast cancer was evaluated using the Breast Cancer Stigma Scale, which employed a 6‐point Likert scale to assess participants′ perceptions of stigma associated with breast cancer [16].

### 2.7. Data Collection

Baseline data were collected prior to intervention implementation using a structured questionnaire that covered demographic details, breast cancer knowledge, prior screening practices, attitudes toward breast cancer, and perceived barriers to screening. Follow‐up data were collected postintervention. Reported breast cancer screening uptake by participants was verified from the records of the mammography facility at the tertiary care center, at a distance range of approximately 40–45 mi, and the records of the mobile mammography van. The structured questionnaire was readministered to assess changes in knowledge, attitudes, and screening behaviors.

### 2.8. Outcome Measures

The primary outcome was the postintervention breast cancer screening uptake/participation rate. Secondary outcomes included changes in breast cancer awareness and stigma reduction. Other outcome measures included an assessment of perceived barriers to screening.

### 2.9. Cost‐Effectiveness Analysis/Input–Output Analysis

An input–output analysis was conducted to assess the cost‐effectiveness of the three intervention models. All inputs were estimated in monetary terms and included expenses related to training, travel, audiovisual aids, stationery, and other logistics. For the medical personnel–led education intervention and the BHV‐led intervention, additional inputs included honoraria for volunteers and out‐of‐pocket expenses borne by participants for screening mammography conducted at the tertiary care hospital. These costs were computed at the end of the intervention period. For the mobile screening mammography van intervention, the primary input was the estimated hiring fee of the van per visit. This estimate encompassed consumables, honoraria for the technician, and reporting fees. The primary outcome across all three arms was defined as the number of mammography screenings completed in each intervention group.

### 2.10. Statistical Analysis

Descriptive statistics were used to summarize baseline demographic characteristics, breast cancer knowledge, and screening practices. The primary outcome, screening uptake, was analyzed using chi‐square tests for categorical variables and *t*‐tests or Mann–Whitney *U* tests for continuous variables. Logistic regression models were employed to control for potential confounders and assess the independent effect of each intervention. Bootstrap resampling was used to derive 95% confidence intervals for ICER, and a one‐way sensitivity analysis assessed robustness by varying key cost inputs ±20%. Secondary outcomes, including changes in knowledge and attitudes, were analyzed using paired *t*‐tests. Qualitative data from participants′ questionnaire responses were analyzed to identify common barriers and suggestions for improvement.

## 3. Results

### 3.1. Participant Demographics, Socioeconomic Status, and Education Level

A total of 944 women from three villages participated in the study, with each village representing one intervention arm. Villages were the unit of randomization; however, outcomes were analyzed at the individual participant level to assess changes in awareness, stigma, and screening uptake. Of these, 331 (35.1%) participants of Village A received education from medical personnel, 297 (31.5%) women of Village B participated in BHV‐led sessions, and 316 (33.5%) of Village C received services of the mobile screening mammography van on‐site visits. Table 1 summarizes participant characteristics. Baseline demographic characteristics were broadly comparable across the three intervention villages. The mean age of participating women was 53.65 (±10.16) years, with an age range of 31—72 years. Most participants were above 50 years of age, with a similar distribution across all three groups. Participants were classified as below the poverty line (BPL) or above the poverty line (APL) based on their socioeconomic status, as defined by national government guidelines. The majority (67.6%) of participants were illiterate or had only primary education. In terms of breast cancer education, 55.0% of participants had received some form of education. Socioeconomic status, education level, and breast cancer education were relatively evenly distributed across the three intervention groups (Table 1).

**Table 1:** Participant demographics, socioeconomic status, education level, and breast cancer education status.

Participant characteristics	Total *N* = 944	Medical personnel–led intervention (*n* = 331)	Breast health volunteer–led intervention (*n* = 297)	Mobile screening mammography van (*n* = 316)
*Age group*				
≤ 50 years	397 (42.1%)	130 (39.3%)	128 (43.1%)	139 (44.0%)
> 50 years	547 (57.9%)	201 (60.7%)	169 (56.9%)	177 (56.0%)
*Socioeconomic status*				
BPL	499 (52.9%)	193 (58.3%)	154 (51.9%)	152 (48.1%)
APL	445 (47.1%)	138 (41.7%)	143 (48.1%)	164 (51.9%)
*Education level*				
Illiterate	316 (33.5%)	125 (37.8%)	109 (36.7%)	82 (25.9%)
Primary	322 (34.1%)	110 (33.2%)	82 (27.6%)	130 (41.1%)
Secondary	171 (18.1%)	58 (17.5%)	55 (18.5%)	58 (18.4%)
Sr. secondary	90 (9.5%)	23 (6.9%)	37 (12.5%)	30 (9.5%)
Graduate	45 (4.8%)	15 (4.5%)	14 (4.7%)	16 (5.1%)
*Cancer education*				
Not educated	425 (45.0%)	147 (44.4%)	136 (45.8%)	142 (44.9%)
Educated	519 (55.0%)	184 (55.6%)	161 (54.2%)	174 (55.1%)

Abbreviations: APL, above poverty line; BPL, below poverty line; Sr., senior.

### 3.2. Breast Cancer Screening Uptake Rate

**Figure 2:**
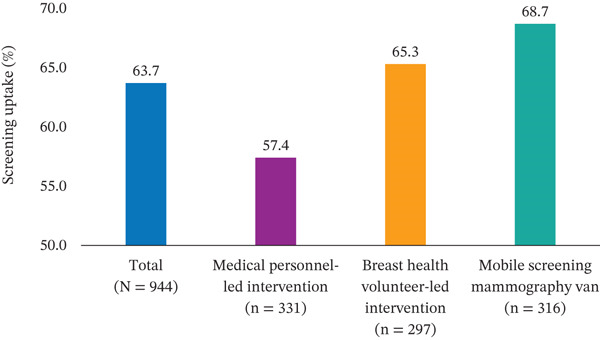
Breast cancer screening uptake. The highest breast cancer screening uptake was noted in the mobile screening mammography van arm (68.7%).

A total of 63.7% (601/944) of participating women underwent screening mammography over the study period. The screening uptake differed significantly across interventions (*χ* ^2^ = 8.93, *p* = 0.003). The highest participation rate of 68.7% was observed in the participants of the mobile screening mammography van group, followed by an uptake rate of 65.3% in the participants of the BHV‐led intervention group. A comparatively lower screening rate of 57.4% was reported among the participants in the medical personnel–led intervention group (Figure 2).

Logistic regression with the medical personnel–led intervention group as reference showed lower odds of uptake in the BHV‐led intervention group (*OR* = 0.62, *p* = 0.003), whereas the mobile screening mammography van group did not differ significantly (*OR* = 0.86, *p* = 0.378). No interactions were observed with age, socioeconomic status, education, or prior breast cancer education. The classification model predicted mammography uptake with accuracy, reflecting the correct classification of those screened but not those unscreened. The mean age of women screened was 53.6 ± 10.3 years (range 21–72), with no significant differences between intervention groups (*F* = 2.57, *p* = 0.078).

### 3.3. Breast Cancer Awareness Score

**Figure 3:**
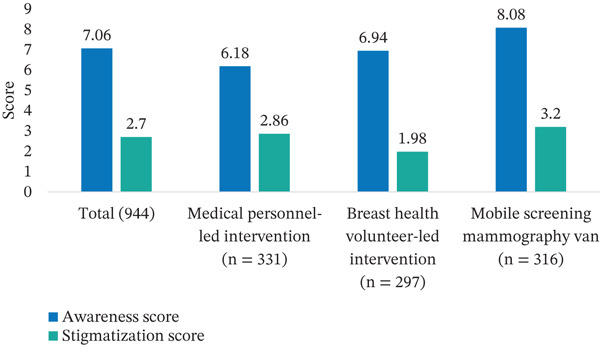
Greatest improvement in awareness score was observed in the mobile screening mammography van‐led group; largest reduction in stigma scores was observed in the BHV‐led intervention group.

Across all groups, awareness scores increased significantly (Table 2). The medical personnel–led intervention group showed a mean postintervention increase of 3.93 ± 1.87 in the awareness score (*p* < 0.0001), whereas the BHV‐led intervention group demonstrated an increase of 4.44 ± 1.91 (*p* < 0.0001). The mobile screening mammography van group exhibited the greatest improvement, with an increase of 5.28 ± 2.41 (*p* < 0.0001) in the awareness score postintervention (Figure 3). Post hoc analysis showed that the increase in breast cancer awareness score was significantly greater in the mobile screening mammography van group than in the medical personnel–led intervention and BHV‐led intervention groups (both *p* < 0.0001).

**Table 2:** Breast cancer awareness scores preinterventions and postinterventions.

Intervention groups	Awareness score		ANOVA test		Paired *t*test
	Pretest	Posttest	*F*	*p* ^∗^	*p* ^∗∗^
Medical personnel–led intervention (*n* = 331)	2.25 ± 1.85	6.18 ± 0.11	83.283	< 0.0001	< 0.0001
Breast health volunteer–led intervention (*n* = 297)	2.49 ± 1.85	6.94 ± 0.12			
Mobile screening mammography van (*n* = 316)	2.80 ± 2.06	8.08 ± 0.09			
Total (*N* = 944)	2.51 ± 1.93	7.06 ± 0.07			

*Note:* Data are presented as *m* *e* *a* *n* ± *S* *D*.

^∗^ *p* < 0.0001 between group comparisons (ANOVA).

^∗∗^ *p* < 0.0001 within group comparisons (paired *t* test).

### 3.4. Reduction in Stigma

All three groups also experienced a significant reduction in stigmatization scores (Table 3). The medical personnel–led intervention group showed a decrease of 1.99 ± 2.71 in the score postintervention (*p* < 0.0001), whereas the mobile screening mammography van group also experienced a reduction of 1.72 ± 2.48 in the score from baseline (*p* < 0.0001) (Figure 3). The BHV‐led intervention group demonstrated the largest reduction in stigmatization score, with a decrease of 2.91 ± 3.56 points postintervention (*p* < 0.0001). Post hoc analysis showed that the reduction in stigmatization score was significantly greater in the BHV‐led intervention group compared with the medical personnel–led intervention and mobile screening mammography van group (both *p* < 0.0001).

**Table 3:** Stigmatization scores preinterventions and postinterventions.

Intervention groups	Stigmatization score		ANOVA test		Paired *t*test
	Pretest	Posttest	*F*	*p* ^∗^	*p* ^∗∗^
Medical personnel–led intervention (*n* = 331)	4.85 ± 2.48	2.86 ± 2.15	26.650	< 0.0001	< 0.0001
Breast health volunteer–led intervention (*n* = 297)	4.88 ± 2.22	1.98 ± 2.00			
Mobile screening mammography van (*n* = 316)	4.92 ± 2.30	3.20 ± 2.25			
Total (*N* = 944)	4.88 ± 2.34	2.70 ± 2.20			

*Note:* Data are presented as *m* *e* *a* *n* ± *S* *D*.

^∗^ *p* < 0.0001 between group comparisons (ANOVA).

^∗∗^ *p* < 0.0001 within group comparisons (paired *t* test).

### 3.5. Perceived Barriers to Screening

**Figure 4:**
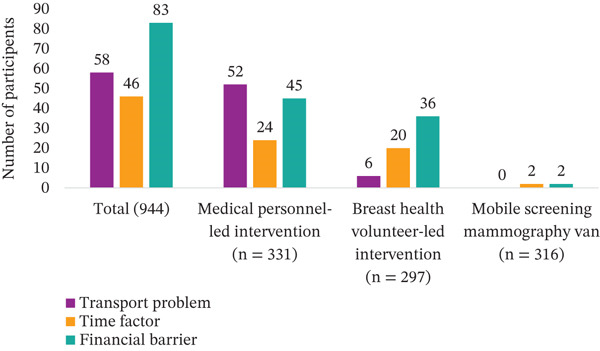
Mobile screening mammography van–led intervention group reported the fewest barriers for breast cancer screening.

Participants reported several barriers to screening, with transport and financial constraints being the most prominent (Table 4). In the medical personnel–led intervention group, 15.7% of the participants reported transport issues, compared with 2.0% in the BHV‐led group and none in the mobile screening mammography van group (*p* < 0.0001). Similarly, time constraints were reported by 7.3% of participants in the medical personnel–led group, 6.7% in the BHV‐led group, and only 0.6% in the mobile screening mammography van group. Financial barriers were noted by 13.6% of participants in the medical personnel–led group, 12.1% in the BHV‐led group, and just 0.6% in the mobile screening mammography van group (*p* < 0.0001) (Figure 4).

**Table 4:** Perceived barriers to screening.

Participant characteristics	Total *N* = 944	Medical personnel–led intervention (*n* = 331)	Breast health volunteer–led intervention (*n* = 297)	Mobile screening mammography van (*n* = 316)	Chi‐square test	
					*χ* ^2^	*p*
Transport problem	58 (6.1%)	52 (15.7%)	6 (2.0%)	0 (0.0%)	21.353	< 0.0001
Time factor	46 (4.9%)	24 (7.3%)	20 (6.7%)	2 (0.6%)		
Financial barrier	83 (8.8%)	45 (13.6%)	36 (12.1%)	2 (0.6%)		

*Note:* A *p* value of < 0.0001 indicates statistical significance.

### 3.6. Adjusted Scores for Awareness and Stigma (*n* = 944)

To account for baseline differences in covariates (age, socioeconomic status, education, prior breast cancer education, and mammography history), a generalized linear model (GLM) with repeated measures was applied. Intervention type was included as the main factor, time (pre vs. post) as the within‐subjects factor, and covariates were entered for adjustment.

All three intervention arms demonstrated substantial increases in awareness from baseline to postintervention (Table 5). In the medical personnel–led education group, adjusted mean awareness scores increased from 2.23 (SE 0.11; 95% CI: 2.03–2.44) at baseline to 6.24 (SE 0.10; 95% CI: 6.04–6.43) postintervention. The BHV‐led group showed an improvement from 2.50 (SE 0.11; 95% CI: 2.28–2.72) to 6.93 (SE 0.11; 95% CI: 6.72–7.13). The mobile screening mammography van group demonstrated the greatest increase, from 2.82 (SE 0.11; 95% CI: 2.60–3.03) to 8.04 (SE 0.10; 95% CI: 7.84–8.24). ANOVA confirmed a significant effect of intervention type on improvement in awareness (*F*(2936) = 45.78, *p* < 0.0001, *η* ^2^ = 0.089, *o* *b* *s* *e* *r* *v* *e* *d* *p* *o* *w* *e* *r* = 1.0). Among covariates, socioeconomic status (*p* = 0.020) and prior mammography (*p* < 0.0001) were also significant predictors.

**Table 5:** Adjusted awareness scores across time points (*n* = 944).

Type of intervention	Time	*N*	Descriptive score (mean±SD)	Adjusted score (mean±SE)	95% CI. for change
Medical personnel–led intervention	Pre	331	2.25 ± 1.85	2.23 ± 0.11	2.03–2.44
	Post	331	6.18 ± 1.99	6.24 ± 0.10	6.04–6.43
Breast health volunteer–led intervention	Pre	297	2.49 ± 1.85	2.50 ± 0.11	2.28–2.72
	Post	297	6.94 ± 2.00	6.93 ± 0.11	6.72–7.13
Mobile screening mammography van	Pre	316	2.80 ± 2.06	2.82 ± 0.11	2.60–3.03
	Post	316	8.08 ± 1.63	8.04 ± 0.10	7.84–8.24

Stigma related to breast cancer decreased significantly across all intervention arms (Table 6). In the medical personnel–led group, adjusted mean stigma scores declined from 4.87 (SE 0.13; 95% CI: 4.61–5.12) to 2.89 (SE 0.12; 95% CI: 2.65–3.12). In the BHV‐led group, scores decreased from 4.88 (SE 0.14; 95% CI: 4.61–5.14) to 1.97 (SE 0.12; 95% CI: 1.73–2.21). In the mobile screening mammography van group, stigma scores dropped from 4.91 (SE 0.13; 95% CI: 4.65–5.16) to 3.18 (SE 0.12; 95% CI: 2.94–3.42). ANOVA revealed a significant effect of intervention type on stigma reduction (*F*(2936) = 10.94, *p* < 0.0001, *η* ^2^ = 0.023, *o* *b* *s* *e* *r* *v* *e* *d* *p* *o* *w* *e* *r* = 0.99). Other baseline covariates were not statistically significant predictors.

**Table 6:** Adjusted stigma scores across time points (*n* = 944).

Type of intervention	Time	*N*	Descriptive score (mean±SD)	Adjusted score (mean±SE)	95% CI. for change
Medical personnel–led intervention	Pre	331	4.85 ± 2.48	4.87 ± 0.13	4.61–5.12
	Post	331	2.86 ± 2.15	2.89 ± 0.12	2.65–3.12
Breast health volunteer–led intervention	Pre	297	4.88 ± 2.22	4.88 ± 0.14	4.61–5.14
	Post	297	1.98 ± 2.00	1.97 ± 0.12	1.73–2.21
Mobile screening mammography van	Pre	316	4.92 ± 2.30	4.91 ± 0.13	4.65–5.16
	Post	316	3.20 ± 2.25	3.18 ± 0.12	2.94–3.42

### 3.7. Cost‐Effectiveness Analysis

Table 7 summarizes the cost‐effectiveness analysis. The medical professional–led intervention had the lowest total cost, resulting in a cost‐effectiveness ratio of INR 810.52 per mammogram. The mobile screening mammography van–based intervention had the highest cost, with a cost‐effectiveness ratio of INR 2304.14 per mammogram.

**Table 7:** Cost‐effectiveness analysis.

Intervention groups	Cost (input) INR	Mammography done (output)	Cost‐effective ratio (input/output)
Medical personnel–led intervention	154,000	190	810.52
Breast health volunteer–led intervention	256,400	194	1321.64
Mobile screening mammography van	500,000	217	2304.14

Calculation of ICER values showed that the additional cost required to achieve one additional screening mammogram when shifting from medical professional–led intervention to BHV‐led intervention was INR 25,600.00. For the switch from medical professional–led intervention to mobile screening mammography van–based intervention, the additional cost was INR 12,814.81. The ICER value between the BHV‐led intervention and the mobile screening mammography van–based intervention was INR 10,591.3. Medical professional–led education emerged as the most cost‐effective tool in terms of improvement in screening mammography scores, followed by the BHV‐led approach.

## 4. Discussion

Breast cancer remains a significant public health challenge in rural areas, where limited awareness, sociocultural barriers, and restricted healthcare access hinder early detection and treatment [10]. Effective educational and screening strategies are essential to reduce the burden of breast cancer and improve overall health outcomes among women in these communities. Several studies have identified that rural women generally lack adequate knowledge about breast cancer and the principles of early detection [9, 17]. Due to poor awareness and inadequate healthcare infrastructure, many cases remain undiagnosed until they reach an advanced stage, contributing to higher mortality rates [9, 17]. Early detection of breast cancer through routine screening significantly reduces morbidity and mortality [18]. Although BSE, clinical breast evaluation, and mammography are established screening methods, uptake remains consistently low in rural communities due to socioeconomic and cultural barriers [19]. Societal stigma surrounding breast cancer remains a major barrier to screening participation and early diagnosis in rural areas [20]. Women often hesitate to seek screening due to fear of diagnosis, misconceptions about cancer, and social stigma associated with the disease [21]. Studies suggest that stigma leads to delays in health‐seeking behaviors, with internalized self‐stigma further discouraging women from participating in screening programs. [22, 23] Perceived barriers, such as financial constraints, transportation difficulties, and limited access to healthcare facilities, significantly affect screening uptake in rural settings [19]. Addressing these challenges and improving breast cancer awareness through targeted educational interventions is critical for enhancing early detection and improving survival rates [24]. Hence, effective educational strategies and screening interventions must be implemented to improve awareness and uptake of breast cancer screening services [10]. Previous research indicates that educational interventions and mobile screening services can significantly increase screening participation [25, 26]. However, the method of education delivery significantly influences its effectiveness in improving breast cancer awareness and screening participation. Our study evaluated three key intervention strategies—medical personnel–led education, BHV‐led education, and mobile screening mammography van intervention—to assess their effectiveness in enhancing breast cancer awareness and screening uptake among rural women.

Dissemination of information through medical personnel is considered more impactful, assuming that their authoritative position ensures adherence. However, medical personnel–led education strategies, though widely accepted, have limitations in rural settings [1]. Despite their proficiency in providing health information, medical personnel are often perceived as outsiders, which can hinder rapport with local communities. This challenge is especially significant in rural areas, where cultural sensitivity and trust are crucial for successful intervention [1]. Although medical personnel are knowledgeable and authoritative figures in healthcare, their outreach efforts often fail to address the sociocultural barriers prevalent in rural communities fully [27–29]. Studies indicate that factors such as limited accessibility to healthcare facilities, financial constraints, and cultural disconnect may hinder the effectiveness of medical personnel‐led interventions in these settings and may not achieve deep community penetration and trust. [27–29] In our study, the medical personnel–led intervention group achieved a screening uptake of 57.4%, which, although lower than the other groups, still represented a significant improvement over the baseline. Although medical personnel–led education emerged as the most cost‐effective tool for improving screening mammography rates, its potential to reduce stigma was inferior to that of other interventions, possibly due to suboptimal engagement with the local community. Our finding highlights the need to complement medical personnel–led education with other interventions, including community engagement and accessible screening services, to enhance its impact.

Cultural beliefs and norms influence women′s perception of breast cancer and their willingness to undergo screening. In many rural areas, discussing breast exams is taboo, leading to low participation. Stigma further prevents women from seeking medical help. Hence, culturally tailored education is essential to increase screening uptake [1]. Community‐participation–based education programs have been increasingly recognized as an effective approach to addressing sociocultural barriers and promoting breast cancer awareness [30]. These programs leverage local educators who are familiar with the community′s cultural context, effectively addressing misconceptions and reducing the stigma associated with breast cancer [30]. By promoting trust and employing culturally sensitive communication strategies, community volunteers can enhance awareness and encourage screening participation [30]. These models of education, where local women are trained to educate their peers, are promising as they draw on the educators′ cultural and social familiarity, making health information more relevant and accessible [31]. Previous studies have demonstrated that community‐participation–based educational programs, when culturally and linguistically tailored, significantly improve knowledge and awareness about breast, cervical, and colorectal cancer screening [32, 33]. These programs have also been reported to increase screening rates more effectively than external medical teams in low‐income and rural settings [1, 34]. Our study found that the BHV‐led intervention group achieved a substantial screening uptake of 65.3% and recorded the most significant reduction in stigma scores. This highlights the importance of community engagement in health interventions, as local educators can build trust and address sociocultural barriers more effectively than external healthcare professionals.

MSUs provide cancer screening services outside fixed clinical settings, thereby increasing access to early detection services [35]. The success of mobile units in rural areas has also been reported previously, demonstrating their ability to overcome logistical barriers by bringing screening services directly to underserved populations [35, 36]. This model is particularly effective in addressing issues of accessibility and affordability, which are significant barriers to cancer screening in rural areas [35, 36]. MSUs have been recognized as a cost‐effective strategy for resource‐limited settings, allowing asymptomatic women who cannot afford to lose their daily wages to access screening services conveniently [37]. By complementing education‐driven interventions with direct clinical services, mobile screening vans enhance participation rates and facilitate early detection [36]. Mobile clinics have been shown to overcome logistical challenges and enhance access to preventive healthcare in rural areas and in low‐resource settings by reducing out‐of‐pocket costs and structural barriers [19, 26, 36]. In our study, the mobile screening mammography van group exhibited the highest screening uptake, indicating that greater on‐site screening accessibility facilitates participation. This model also successfully mitigated barriers such as transportation costs and financial constraints, making it a highly effective strategy for rural populations.

Overall, the study demonstrated that all three intervention strategies effectively improved breast cancer awareness, screening participation, and stigma reduction. The mobile screening mammography van group demonstrated the highest posttest awareness scores, suggesting that direct engagement combined with screening services enhances knowledge retention and encourages proactive health behavior. Our study found that the BHV‐led intervention was the most effective in reducing stigma, likely due to its culturally sensitive, peer‐driven approach. Addressing stigma through community‐based strategies can empower women to seek timely medical attention without fear of social judgment. Our findings align with published literature, indicating that transport and financial constraints were most prominent in the medical personnel–led education group and least reported in the mobile screening mammography van group. This suggests that mobile health interventions effectively address common barriers by providing convenient and cost‐effective access to screening services. The highest screening uptake in the mobile screening mammography van group reinforces the effectiveness of this approach in overcoming logistical constraints.

This study has a few limitations. First, it was conducted in a specific rural region of the country, which may limit the generalizability of the findings to other populations with different healthcare infrastructures and sociocultural contexts. Second, the study relied on self‐reported data for certain aspects, which may introduce recall bias and social desirability bias, potentially affecting the accuracy of responses regarding awareness and stigmatization. Third, while the interventions were assessed over 2 years, long‐term behavioral changes and sustained screening adherence were not evaluated. Importantly, this was a cluster‐based study with only three clusters. Analyses were performed at the individual level, which may not have fully accounted for intracluster correlation. The small number of clusters introduces a potential risk of Type I error, and the findings should therefore be interpreted with caution. Additionally, there was variability in participation rates across the intervention groups, which might have influenced the outcomes. External factors and broader socioeconomic influences were not comprehensively analyzed and may have affected the study results. Future research should incorporate larger, more diverse populations and longitudinal follow‐ups to assess the lasting impact of these intervention strategies.

## 5. Conclusion

Our study highlighted the effectiveness of mobile screening mammography units and BHV‐led initiatives in improving breast cancer awareness, increasing screening uptake, and reducing stigma among rural women. These approaches address critical barriers, including accessibility, affordability, and cultural misconceptions. Implementing integrated multicomponent interventions that combine community engagement with MSU deployment can substantially improve breast cancer awareness and early detection in rural settings.

## Funding

No funding was received for this manuscript.

## Conflicts of Interest

The authors declare no conflicts of interest.

## Data Availability

The data that support the findings of this study are available on request from the corresponding author. The data are not publicly available due to privacy or ethical restrictions.
